# HTLV infection in urban population from Mato Grosso do Sul, Central Brazil

**DOI:** 10.1186/s12977-024-00650-1

**Published:** 2024-11-05

**Authors:** Carolina Amianti, Larissa Melo Bandeira, Wesley Marcio Cardoso, Andréia Souza Pinto da Silva, Milena da Silva de Jesus, Rodrigo Ibañez, Felipe Bonfim Freitas, Silvia Naomi de Oliveira Uehara, Izaura Maria Vieira Cayres Vallinoto, Antonio Carlos Rosário Vallinoto, Ana Rita Coimbra Motta-Castro

**Affiliations:** 1 Universidade Federal de Mato Grosso do Sul, Campo Grande, Mato Grosso do Sul Brazil; 2 Secretaria Municipal de Saúde de Campo Grande, Campo Grande, Mato Grosso do Sul Brazil; 3 Instituto Evandro Chagas, Ananindeua, Pará Brazil; 4 Universidade Federal do Pará, Belém, Pará Brazil

**Keywords:** HTLV, Prevalence, Central Brazil

## Abstract

**Background:**

Brazil has the highest number of HTLV-1 infection in Latin America, with around one million cases spread unevenly across regions. However, there is a limited number of studies on this infection in the general population. This cross-sectional study aimed to estimate the prevalence of HTLV as well as identify types, and subtypes of HTLV among the urban population of Campo Grande, capital of Mato Grosso do Sul state (MS).

**Results:**

Between July 2023 and March 2024, all information was obtained from self-reported interviews, and blood samples were collected and screened for anti-HTLV-1/2 by immunoassay and confirmed using the immunoblot method. The proviral DNA of HTLV-1/2 in positive samples was quantified by real-time PCR (qPCR) and genotyped by nucleotide sequencing (Sanger’s method). The study enrolled 611 participants, with the majority being women (90.54%), mixed race (46.32%), heterosexual (87.64%), and with a median age of 39 years. The prevalence rate of anti-HTLV-1 infection was 0.82% (CI 95% 0.34–1.96). All positive samples (n = 5) were identified as belonging to the Cosmopolitan subtype, one belonging to Japanese and four to Transcontinental subgroups. Among the five positive individuals, two presented symptoms associated with HTLV-1 infection.

**Conclusion:**

This study highlights an intermediate prevalence of HTLV-1 in the urban population of Campo Grande, Mato Grosso do Sul, and provides epidemiological information that could help bridge the gaps in the distribution of HTLV in the general population. Also, medical care was provided for individuals presenting clinical manifestations who were previously unaware of their serological status.

## Introduction

Human T-lymphotropic virus (HTLV) is a retrovirus that mainly targets T lymphocytes. This includes HTLV-1 and HTLV-2, which were identified in the 1980s, and later, HTLV-3 and HTLV-4 [1, 2]. HTLV-1 is categorized into seven distinct subtypes: 1-a to 1-g. The 1-a or Cosmopolitan subtype (HTLV-1a), is divided into six subgroups [3]. Of these, the Japanese (HTLV-1aJpn) and Transcontinental (HTLV-1aTC) subgroups have already been reported in Brazil [4–8].

The routes of transmission include contact with contaminated blood products, vertical transmission, and condomless sexual intercourse [9]. HTLV is associated with the development of several diseases such as HTLV-1-associated myelopathy (HAM), adult T-cell leukemia/lymphoma (ATLL), infectious dermatitis, uveitis, arthritis, and other “minor” neurological signs that do not meet diagnostic criteria for HAM [10].

HTLV-1 is prevalent globally, notably in regions such as South Japan, the Caribbean Basin, and parts of South America. Brazil has the largest number of people living with HTLV-1 in Latin America, with approximately one million people infected heterogeneously distributed throughout all regions [2, 11].

Several studies have reported the presence and prevalence of HTLV infection among specific groups in Brazil [7, 8, 12–18]. Furthermore, the introduction of mandatory HTLV-1/2 screening in potential blood donors in 1993 allowed for a better understanding of the distribution of the virus in all regions of the country [19, 20]. However, blood donors and vulnerable groups in Brazil may not represent the general population due to various factors such as age restrictions, gender disparities, risk behaviors, and health conditions.

This cross-sectional study aimed to describe the prevalence of HTLV infection in the urban population of Campo Grande, the capital of Mato Grosso do Sul state, and the seroepidemiological and molecular profiles of HTLV-1/2.

## Materials and methods

### Ethics statement

This study was approved by the Ethical Committee on Human Research of the Universidade Federal de Mato Grosso do Sul (CEP/UFMS) by protocol number CAAE: 63756922.00000.0021. All research was performed according to relevant guidelines and regulations. The consent form elucidated the study's objectives, voluntary nature of participation, and right to withdraw at any time without repercussions. Participants were informed about the procedures, including blood sample collection and the confidentiality of their data. It was explicitly stated that if they tested positive for HTLV-1/2, they would receive their results privately via email or telephone, accompanied by appropriate medical referrals, if warranted. Furthermore, participants were informed of potential health implications and the importance of early detection, and were provided with information regarding prevention and treatment of symptoms options. This protocol ensured that all participants were aware of their rights, nature of the study, and consequences of a positive result.

### Sample calculation

The calculation of the sample size for the number of individuals to be investigated was defined by the calculation of prevalence in infinite populations, as there was no assumption of prevalence estimated for HTLV. A maximum frequency of 50% was used to obtain the largest sample size with a confidence level of 95%, sampling error of 2%, and non-response rate of 20%. This cross-sectional study was a part of a population-based study conducted in the northern, northeastern, and central-western regions of Brazil. Therefore, for the study in Mato Grosso do Sul State, a sample size of 319 individuals was considered the minimum number of individuals participating in the study.

### Study population

A cross-sectional study was conducted among the urban population of Campo Grande, capital of Mato Grosso do Sul state (MS)—Central Brazil, between July 2023 and March 2024. The general profile of residents from the capital is, in the majority, mixed race, median age of 34 years, and women [21]. The inclusion criteria were residents of the capital city with a minimum age of 9 years. Prior to enrollment, all study participants or, in the case of individuals under the age of 18, their legal guardians provided written informed consent. Participants underwent an interview with a standardized questionnaire containing sociodemographic and risky behavior information and provided blood samples.

**Fig. 1:**
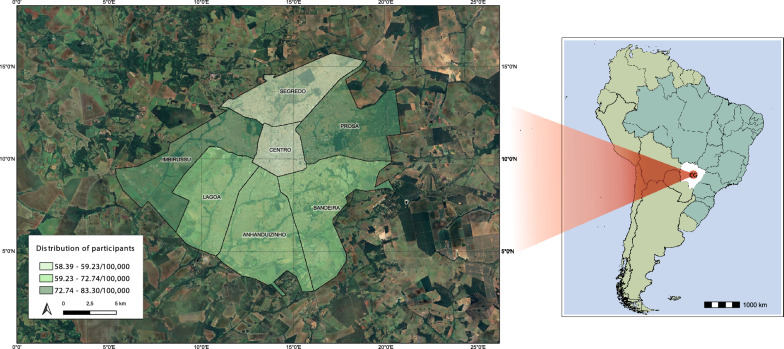
Distribution of participants from all seven sanitary districts in Campo Grande (CG), the capital of MS State. To calculate the distribution, the total number of participants in the district/total number of residents in district X 100,000. The map was generated using QGIS (version 3.26.2) with 3 classes precision, and the Jenks natural breaks model was selected.

This study was conducted using a convenience method at the Universidade Federal de Mato Grosso do Sul, and 21 basic family health units were distributed in all seven sanitary districts in the capital (Fig. 1). These health units carry out health promotion, protection, and recovery activities, ranging from administering vaccines and dispensing medications to dental care.

### Serological and molecular tests

All samples were screened using a commercial enzyme-linked immunosorbent assay (ELISA) kit (Murex HTLV I + II—DiaSorin) following the manufacturer’s instructions to detect anti-HTLV-1/2 antibodies. Positive samples were repeatedly tested and confirmed using an HTLV-1/2 Western Blot (WB) assay (MP Diagnostics HTLV BLOT 2.4, Singapore). HTLV infection was defined as positive in both ELISA screening and confirmatory WB tests.

The anti-HTLV-positive samples were subjected to molecular analysis. To determine proviral load, peripheral blood mononuclear cells (PBMCs) were isolated from EDTA blood samples of HTLV-1-infected individuals using density gradient centrifugation. DNA was extracted using the QIAamp DNA Blood Mini Kit (Qiagen) and subjected to amplification by multiplex real-time PCR (mqPCR) with an amplicons size of 100 bp of the HTLV-1/2 tax region to determine proviral load, as previously reported [22].

For sequencing, DNA was extracted from whole blood samples of anti-HTLV-positive participants using the QIAamp DNA Blood Mini Kit (Qiagen), according to the manufacturer’s instructions. A 646 bp fragment of the HTLV-1 5’LTR region was amplified by nested PCR [23]. The amplicons were purified using a QIA Quick Purification kit (Qiagen Inc., Maryland, USA) according to the manufacturer’s instructions. The fragments were sequenced by Sanger's method using the BigDye Terminator Cycle Sequencing Ready Reaction Kit and ABI 3500XL (Applied Biosystems, Foster City, CA, United States).

HTLV-1 isolates were subjected to analysis using the Basic Local Alignment Search Tool (BLAST) after nucleotide sequencing to identify subtypes and subgroups. Nucleotide sequences were aligned and compared to 55 published HTLV-1 sequences available from GenBank, with reference sequences used as outgroups, and sequences were searched with filter HTLV-1 and Transcontinental and Brazil, using MAFFT plugin, version 7.308, available in Geneious software, version 7.1.3, employing the L-INS-i algorithm [24].

Phylogenetic tree inference analysis was reconstructed using Maximum Likelihood analysis and performed using RAxML, utilizing the GTRGAMMA model. Support values were estimated from 1000 bootstrap pseudoreplicates on the CIPRES Science Gateway portal (https://www.phylo.org) [25]. The phylogenetic tree was edited using the Interactive Tree Of Life (iTOL) software v6 (https://itol.embl.de) [26].

GenBank nucleotide sequence accession number included in the phylogenetic analysis: DQ235700; L36905; MK936943; KM023768; KM023765; KM023769; KM023757; AF054627; Y16481; DQ235698; DQ235699; J02029; JF271837; JF271838; AF033817; DQ070891; DQ070892; DQ471196; FJ853491; KY490575; FJ853490; DQ471194; DQ471193; OM863789; OM863790; OK247616; OK247617; KY510690; KY490581; JF271836; GQ443755; KM023763; KM023762; GQ443757; JF271840; JF271842; JF271841; KY510691; GQ443756; DQ471187; DQ471195; DQ471190; DQ471192; DQ471191; DQ471189; DQ471188; EU392160; AY499185; EU392159; Y17014; AY920503; Z32527; L76310; DQ471197; L02534.

### Data analysis

The variables were analyzed using the Stata software (version 13.0; Stata Corporation, College Station, TX, USA). Categorical variables were presented as absolute and percentage frequencies. Continuous variables were expressed as medians and ranges. The prevalence of anti-HTLV-1/2 was calculated with a 95% confidence interval (95% CI). Fisher’s exact test was used to evaluate the sociodemographic and behavioral characteristics of HTLV-1/2 (p < 0.05 were considered statistically significant).

## Results

A total of 611 individuals agreed to participate in the study between July 2023 and March 2024 (Table 1) from all seven sanitary districts from the Capital, with participation coverage ranging from 58.39 to 83.30/100,000 from each district (Fig. 1). The general profile of the group based on self-report was a majority of women (90.54%), with a median age of 39 years (1IQ 28 to 3IQ 54 years old). Most of them considered themselves to be mixed race (46.32%), 87.64% heterosexual, and 99.67% cisgender. In addition, most of them (49.67%) had a high educational level (> 12 years of study), and a low family income (42.76%), with one up to two minimum wages ($660). Furthermore, 62.14% were born by normal delivery, and 90.76% were breastfed, with a majority for more than 6 months (76.54%) by their biological mother (93.78%). Most of the study participants declared that they were not undergoing any health monitoring during the recruitment period (56.07%). Of those who underwent any medical follow-up, eight individuals were related to Sexually Transmitted Infections (STIs).

**Table 1:** Sociodemographic and behavioral characteristics of urban population, Campo Grande-MS (n = 611)

Variable	Negative (n = 606)		Positive (n = 5)		p value
	n	%	n	%	
Age (years)					
< 45	366	99.46	2	0.54	0.861
≥ 45	240	98.77	3	1.23	
Biological sex					
Male	179	99.44	1	0.56	0.539
Female	427	99.07	4	0.93	
Sexual orientation					
Heterosexual	528	99.25	4	0.75	0.484
Homosexual	43	97.73	1	2.27	
Others	31	100	0	0	
Marital status					
Steady partner	290	98.98	3	1.02	0.462
No steady partner	316	99.18	2	0.63	
State of birth in Brazil					
Mato Grosso do Sul	436	99.32	3	0.68	0.624
Other states	170	98.80	2	1.16	
Ethnicity					
White	239	99.17	2	0.83	0.086
Mixed race	282	99.65	1	0.35	
Black	70	98.59	1	1.41	
Japanese descendant	9	90.00	1	10.00	
Others	6	100	0	0	
Years of study					
> 12	302	100	0	0	0.064
10–12	191	98.45	3	1.55	
1–9	99	98.02	2	1.98	
lecture	11	100	0	0	
History of tattoo					
No	401	99.26	3	0.74	0.550
Yes	205	99.03	2	0.97	
History of blood transfusion before 1993					
No	555	99.46	3	0.54	0.067
Yes	53	96.36	2	3.64	
Ever exposed to blood in accidents					
No	521	99.24	4	0.76	0.480
Yes	72	98.63	1	1.37	
History of condom use (lifetime)					
Regular	122	100	0	0	0.317
Irregular	470	98.95	5	1.05	
History of STI					
No	477	99.17	4	0.83	0.592
Yes	93	98.94	1	1.06	
Familiar PLwHTLV					
No	320	99.38	2	0.62	0.924
Yes	13	100	0	0	
Breastfeeding historical					
No	51	100	0	0	0.747
Yes	498	99.40	3	0.60	
Breastfed by					
Biological mother	449	99.34	3	0.66	0.824
Cross-nursing	30	100	0	0	

STI: sexually transmitted infection; PLwHTLV: People living with HTLV

**Fig. 2:**
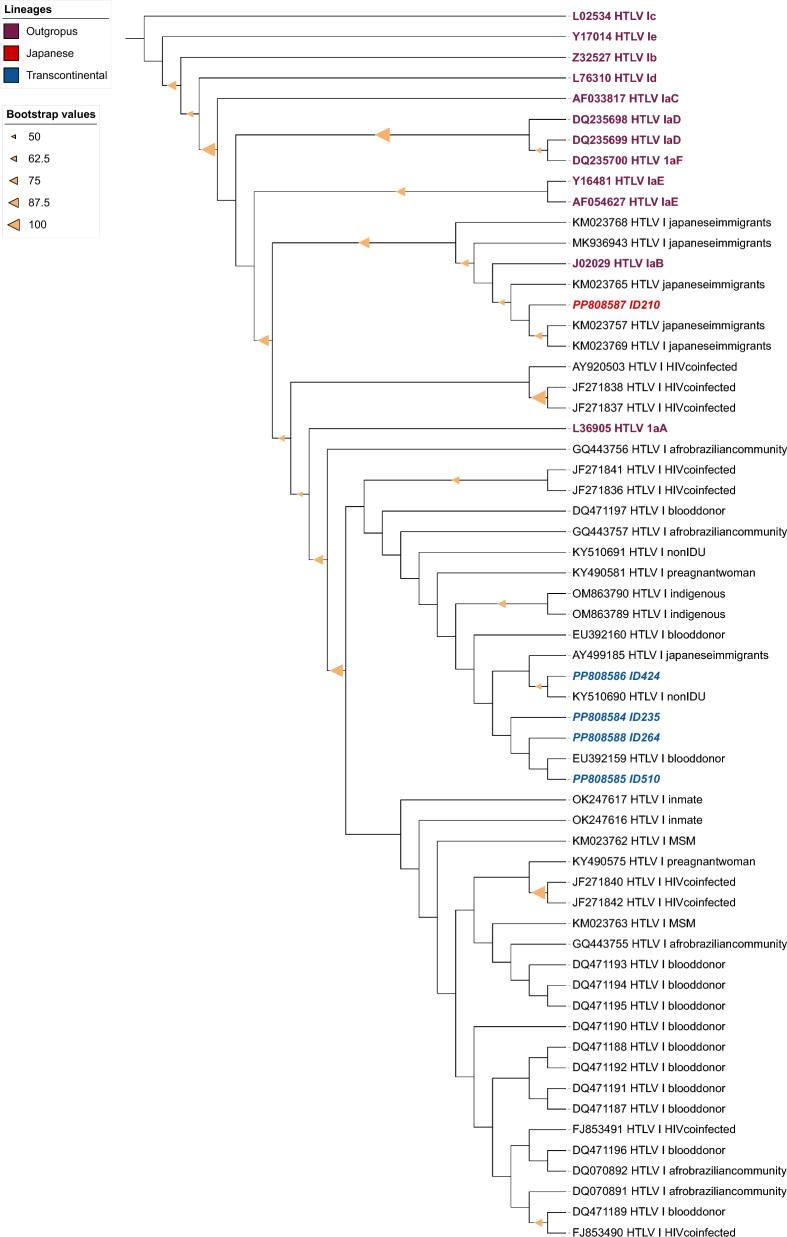
Phylogenetic tree analysis. Phylogenetic tree of HTLV-1 subtypes from different groups. Phylogenetic tree constructed based on Maximum likelihood. Support for branching was determined by 1000 bootstrap replicates, and only values of 50% or higher were shown for Maximum likelihood. PP808584 (ID-235), PP808585 (ID-510), PP808586 (ID-424), PP808587 (ID-210), PP808588 (ID-264) clustered with different subgroups (Japanese and Transcontinental) and population groups.

Five individuals were screened and confirmed as anti-HTLV-1 seropositive. We did not identify any familial connections among positive individuals. Furthermore, the prevalence of anti-HTLV-1 found was 0.82 (CI 95% 0.34–1.96). All five positive samples were successfully sequenced. The phylogenetic analysis revealed that isolates were classified as Cosmopolitan subtype (HTLV-1a), one Japanese (HTLV-1aJpn) and four Transcontinental (HTLV-1aTc) subgroups (Fig. 2), and the GenBank accession numbers for these sequences were: PP808584 (ID-235), PP808585 (ID-510), PP808586 (ID-424), PP808587 (ID-210), and PP808588 (ID-264). Four positive samples were amplified by mqPCR to determine HTLV DNA proviral load (PVL), and the results varied from 253 to 21,876 copies per 10^6^ cells (Table 2).

**Table 2:** Sociodemographic, risk behavior, and virological aspects of 5 individuals anti-HTLV-1 positive

Characteristic	ID-210	ID-235	ID-264	ID-424	ID-510
Sanitary district	Imbirussu	Anhanduizinho	Anhanduizinho	Bandeira	Imbirussu
Type of HTLV	HTLV-1	HTLV-1	HTLV-1	HTLV-1	HTLV-1
Subtype	Cosmopolitan	Cosmopolitan	Cosmopolitan	Cosmopolitan	Cosmopolitan
Subgroup	Japanese	Transcontinental	Transcontinental	Transcontinental	Transcontinental
Presence of symptoms related to HTLV-1 infection	Asymptomatic	Asymptomatic	Symptomatic	Symptomatic	Asymptomatic
HTLV DNA Proviral load (copies/10^6^ cells)	2255	253	21,876	404	Sample not provided
Age (years)	67	65	42	77	29
Biological sex	Male	Female	Female	Female	Female
Ethnicity	Japanese descendant	Black	White	Mixed race	White
History of STI	No	No	Yes	No	No
History of condom use (lifetime)	Irregular	Irregular	Irregular	Irregular	Irregular
Blood transfusion before 1993	No	Yes	No	No	No
History of IDU	No	No	No	No	No
Family history of PLwHTLV	Do not know	Do not know	No	No	Do not know

STI: sexually transmitted infection; IDU: Injecting Drug Use; PLwHTLV: People living with HTLV

All individuals who were anti-HTLV-1 positive were referred to an infectious disease specialist for clinical evaluation at the University Hospital, but only three participants agreed, while the other two did not attend the clinical evaluation due to a lack of interest, even in our effort to refer them to clinical follow-up. During the interview, ID-210 reported that he had been monitored for herpes zoster and did not have any risk behavior for HTLV, except for a history of irregular condom use. Upon clinical evaluation, a characteristic scar in the left cervical region and left hemithorax region was observed. No signs or symptoms of HAM were identified through physical examination (grade 2 patellar reflex, no clonus, and no muscle spasticity). ID-264 self-reported history of HPV infection, urinary incontinence, and muscle and joint pain. During the medical evaluation, no signs or symptoms of HAM were observed. However, tendonitis and bursitis in the left shoulder, mixed depression, and anxiety disorder with cognitive impairment were also present. ID-424 reported experiencing dry eyes and mouth, as well as urinary incontinence, muscle pain, numbness in her lower limbs, joint pain, and difficulty walking following a car accident in 2009, which resulted in her use of a wheelchair. Although no signs of uveitis were present in medical care, neurogenic bladder and bilateral spastic paraparesis were identified, according to Castro Costa [27]. After clinical evaluation, it was determined that the traffic accident in which the participant was involved was not the cause of her difficulty walking, but rather an HTLV-1-associated disease. The ID-235 reported a history of blood transfusion before 1993 and irregular condom use. Meanwhile, ID-510 informs have piercing and irregular condom use. Neither ID-235 nor ID-510 attended the medical evaluation and did not report any signs or symptoms related to HTLV. Among all HTLV-1-positive individuals, two had symptoms related to infection.

## Discussion

Investigations into the prevalence of HTLV infection in the state of Mato Grosso do Sul were performed specifically in groups of the Japanese community, pregnant women, blood donors, the indigenous population, prisoners, men who have sex with men, and remnant *quilombos*, with rates varying from 0.1% to 10.0% [7, 13–15, 28–31]. Therefore, this is the first HTLV study to be carried out in the state of Mato Grosso do Sul in an urban area, covering the general population and, to the best of our knowledge, in Central Brazil. The high coverage of participation shows a strong commitment and interest from the community in contributing to the research, bolstering the validity and representativeness of the findings (Fig. 1). The sociodemographic profile of all study group corroborates with the scenario of the residents from Campo Grande, majority of habitants are mixed race, median age of 34 years old, and women [21]. Regarding the sample containing a higher proportion of women, this reflects the actual demographic composition of the city, as indicated by the Brazilian Institute of Geography and Statistics (IBGE) [21], which reports that there are more women than men in the population of Campo Grande. Furthermore, in addition to the significant increase in the number of women in the study, it is important to note the Brazilian cultural context in which women tend to assume a more proactive role in healthcare than men. Social and cultural norms attribute to women the responsibility of caring for the family, which often extends to their own health.

The prevalence of HTLV-1 infection was 0.82% (CI 95% 0.34–1.96). It can be stratified as low to medium [32] if the CI interval is considered. A study conducted in a metropolitan area in Belém found a prevalence of 1.40%, considering only HTLV-1 [33]. More recently (2022), another study conducted in the same geographic area found a prevalence of 0.38% (CI 0.07–0.69%) for HTLV-1/2 and 0.19% for HTLV-1 [34]. Accordingly, this indicates a decrease in the prevalence in Belém area, which may be attributed to the sample selection process and, also the alteration in the sensitivity of diagnostic techniques. The results of our study are consistent with those of the first investigation, which revealed an intermediate prevalence rate. Additionally, our findings indicate a higher prevalence of HTLV-1 in Belém compared to the current scenario.

Prevalence studies among blood donors have often led to extrapolation of the findings to the general population due to the lack of studies in this one. Studies varied in their rates, ranging from 0.17 to 4.8/1000 donations [31, 35–37], with an estimated global prevalence in the country of 0.4% [38]. However, owing to the risk-free behavioral selection process for candidate blood donors, this could potentially reduce the global prevalence of PLwHTLV in the general population.

All five HTLV-positive individuals were tested for type 1 of the virus, which is consistent due to South America being an endemic region for HTLV-1. It is expected that HTLV-2 would be found in restricted groups such as PWID (Person Who Inject Drugs) and indigenous communities [2]. However, even in indigenous populations of MS State, only the presence of HTLV-1 has been reported [30]. The study found Japanese (HTLV-1aJpn) and Transcontinental (HTLV-1aTc) subgroups belonging to the Cosmopolitan subtype. The Japanese subgroup (HTLV-1aJpn) has previously been identified on both national territory and in MS State [4, 7, 8], as well as within the transcontinental subgroup [15, 30, 39, 40].

HTLV DNA Proviral load (PVL) could be divided into three categories based on the percentage of HTLV-1 DNA copies per 10^**2**^ PBMC: < 1% as low, 1–10% as medium, and > 10% as high [41]. Several hypotheses can be proposed regarding the PVL results obtained in this study, ranging from 253 to 21,876 copies per 10^6^ cells (PBMC).

First, it should be noted that PVL levels cannot be directly compared between participants, as both symptomatic and asymptomatic individuals living with HTLV may have high or low PVL levels [42, 43]. Therefore, this assessment seems to be more useful as a prognostic factor for patient follow-up than for comparative purposes. Second, the PVL of ID-264, 21,876 (copies/ 10^**6**^ cells) may be related to the cognitive impairment observed, as there is a correlation between elevated PVL and neuropsychological deviations [44]. Third, the PVL of ID-424, 404 (copies/106 cells) as a symptomatic carrier may be explained by an A125 mutation in tax-responsive elements, which has been reported in Latin American strains of the HTLV-1 Cosmopolitan Transcontinental subtype. This mutation is associated with lower PVL without affecting the progression of HTLV-1-related diseases [45]. These cases suggest that while there is a potential association between proviral load and clinical presentation, the relationship is complex and may involve other factors, such as genetic mutations.

It is worth mentioning here that, in the case of ID-424, it took more than 10 years to receive a correct diagnosis of HTLV-1, during which time the patient's symptomatology was misattributed to a traffic accident by medical professionals. It is crucial to emphasize the need for ongoing education and training of all healthcare professionals. The delay in obtaining an accurate diagnosis may have affected the patient’s current clinical condition. During the data collection phase, health promotion actions were conducted with the general population and healthcare professionals, and informative leaflets on HTLV were distributed, providing clarification about the infection, transmission routes, associated diseases, necessary examinations, and the importance of diagnosis. In addition to knowing her HTLV serological status and the cause of her leg symptoms, patient ID-424 was referred and has been followed up in multidisciplinary medical care and started physiotherapy.

The current study had certain limitations that must be acknowledged. Underreporting of risk behaviors may have occurred due to fear and distrust associated with discrimination and stigma as well as memory bias, which could have resulted in participants forgetting or misremembering relevant events. These factors could lead to an underestimation of the potential risk factors associated with HTLV infection. Additionally, over-representation of certain demographic groups was observed. These limitations are inherent to the design of this study.

This study presents a comprehensive view of the urban population in the capital city, as it employed a significant sample size that was representative of the population. Additionally, this study provides epidemiological information that could help bridge gaps in the distribution of HTLV infection in the general population, as the results indicate the presence of HTLV-1 infection in Campo Grande. The data obtained can be used to develop effective public health programs aimed at reducing viral transmission. Overall, these findings underscore the importance of the epidemiological scenario of HTLV infection in the general population residing in urban areas.

We propose expanding investigations to additional regions, particularly those with limited healthcare infrastructure or restricted access to medical services, where a lack of awareness regarding infection may be of concern. Furthermore, it emphasizes the significance of continuous monitoring of HTLV-1 epidemiology in diverse settings to enhance control and prevention strategies.

## Data Availability

No datasets were generated or analysed during the current study.
